# Improved Feature-Selection Method Considering the Imbalance Problem in Text Categorization

**DOI:** 10.1155/2014/625342

**Published:** 2014-05-26

**Authors:** Jieming Yang, Zhaoyang Qu, Zhiying Liu

**Affiliations:** College of Information Engineering, Northeast Dianli University, Jilin, Jilin 132012, China

## Abstract

The filtering feature-selection algorithm is a kind of important approach to dimensionality reduction in the field of the text categorization. Most of filtering feature-selection algorithms evaluate the significance of a feature for category based on balanced dataset and do not consider the imbalance factor of dataset. In this paper, a new scheme was proposed, which can weaken the adverse effect caused by the imbalance factor in the corpus. We evaluated the improved versions of nine well-known feature-selection methods (Information Gain, Chi statistic, Document Frequency, Orthogonal Centroid Feature Selection, DIA association factor, Comprehensive Measurement Feature Selection, Deviation from Poisson Feature Selection, improved Gini index, and Mutual Information) using naïve Bayes and support vector machines on three benchmark document collections (20-Newsgroups, Reuters-21578, and WebKB). The experimental results show that the improved scheme can significantly enhance the performance of the feature-selection methods.

## 1. Introduction

Text categorization [1], which assigns the predefined categories to an unlabeled text document [2], has become a very efficient method to manage the vast volumes of digital documents available on the Internet. In recent years, many sophisticated machine learning algorithms, such as support vector machine (SVM) [3], naïve Bayes (NB) [4], and K-nearest neighbor (KNN) [2, 5], have been extensively applied to the text categorization.

The high dimensionality is the major characteristic of text categorization in which the number of the features can easily reach the orders of tens of thousands even for moderate size dataset [6, 7]. Most of features are irrelevant and lead to poor performance of the classifier [8]. Therefore, the dimensionality reduction, which attempts to reduce the size of feature space without sacrificing the performance of the text categorization, has been a critical step in text categorization [1, 9]. Feature selection [10], which selects a subset from original feature space according to evaluation criteria, is the most commonly used dimensionality reduction method in the field of the text categorization [11]. Feature-selection methods can be divided into three classes [12]. One is the embedded approach that the process of the feature selection is embedded in the induction algorithm; another one is the wrapper approach that the evaluation function is used to select the feature subset as a wrapper around the classifier algorithm [11, 13, 14]; the last one is the filtering approach that the evaluation function used to select the feature subset is independent of the classifier algorithm [14]. In this paper, we focus on the filtering approach. Many efficient and effective filtering feature-selection methods have been applied to text categorization, such as Information Gain (IG) [7], Chi-square statistics (CHI) [7, 15], Mutual Information (MI) [16], Document Frequency (DF) [7], improved Gini index (GINI) [17], DIA association factor (DIA) [1, 6], Comprehensive Measurement Feature Selection (CMFS) [11], Orthogonal Centroid Feature Selection (OCFS) [18], and Deviation from Poisson Feature Selection (DFPFS) [15].

So far, almost all of feature-selection algorithms evaluate the significance of a term based on the balanced datasets without considering the influence of the imbalanced factor. In fact, most of data in the real world is imbalanced. There are two reasons why there exist the imbalanced data in the world. One is the intrinsic nature of such event; the rare events yield less samples. The other reason is the expense of collecting samples and legal or privacy reasons [19]. The imbalanced factors in the datasets degrade the performance of the learning algorithms [20]. In recent years, the imbalanced learning problem has got broad attention of numerous experts and scholars [21–23]. In this paper, an improved scheme of existing feature-selection methods is proposed, which weakens the influence of the imbalanced factors occurring in the dataset. In our experiments, we applied the improved scheme on NB and SVM using three benchmark corpora. We favorably show the effectiveness of our approach by demonstrating that it significantly outperforms nine existing feature-selection algorithms.

The rest of this paper is organized as follows.  Section 2 presents nine existing feature-selection algorithms used in the paper.  Section 3 describes the basic idea and implementation of the improved scheme of nine existing feature-selection methods. The experimental details are given in Section 4 and the experimental results are presented in Section 5.  Section 6 shows the statistical analysis and discussion. Our conclusion and the future work direction are provided in the last section.

## 2. Related Feature-Selection Algorithms

### 2.1. Information Gain (IG)

Information Gain [24] is a criterion commonly used in the machine learning [7]. The Information Gain of the feature *t*_*k*_ over the class *c*_*i*_ is the reduction in uncertainty about the value of *c*_*i*_ when the value of *t*_*k*_ is known. The Information Gain of the feature *t*_*k*_ over the class *c*_*i*_ can be calculated as follows:

(1) $$\begin{array}{c} \text{IG}\left(t_{k},c_{i}\right)=\underset{c\in \left\{c_{i},{\overset{-}{c}}_{i}\right\}\;}{\sum }\underset{t\in \left\{t_{k},{\overset{-}{t}}_{k}\right\}}{\sum }P\left(t,c\right)\log\frac{P\left(t,c\right)}{P\left(t\right)P\left(c\right)}, \end{array}$$

where *P*(*c*) is the fraction of the documents in category *c* over the total number of documents and *P*(*t*, *c*) is the fraction of documents in the category *c* that contain the word *t* over the total number of documents. *P*(*t*) is the fraction of the documents containing the term *t* over the total number of documents [25].

### 2.2. Chi-Square (CHI)

Chi-square testing [7] was applied to evaluate the independence of two variables in mathematical statistics. In this paper, the independence of the feature *t*_*k*_ and the category *c*_*i*_ is measured by Chi-square. The greater the value of the CHI(*t*_*k*_, *c*_*i*_) is, the more category information the feature *t*_*k*_ contains. Chi-square formula is defined as follows:

(2) $$\begin{array}{c} \text{CHI}\left(t_{k},c_{i}\right)=\frac{N{\left(a_{ki}d_{ki}-b_{ki}c_{ki}\right)}^{2}}{\left(a_{ki}+b_{ki}\right)\left(a_{ki}+c_{ki}\right)\left(b_{ki}+d_{ki}\right)\left(c_{ki}+d_{ki}\right)}, \end{array}$$

where *N* is the amount of documents in the training set; *a*_*ki*_ is the frequency with which feature *t*_*k*_ occurs in the category *c*_*i*_; *b*_*ki*_ is the frequency with which feature *t*_*k*_ occurred in all categories except *c*_*i*_; *c*_*ki*_ is the frequency with which category *c*_*i*_ occurs and does not contain feature *t*_*k*_; *d*_*ki*_ is the number of times neither *c*_*i*_ nor *t*_*k*_ occurs.

### 2.3. Mutual Information (MI)

Mutual Information is a concept in information theory, which measures the dependencies between random variables and can be applied to measure the information content contained in a feature [26]. Mutual Information is used to measure the dependence between the feature *t*_*k*_ and the category *c*_*i*_ in the feature selection. The higher Mutual Information with the category *c*_*i*_ the feature *t*_*k*_ possesses, the more information about category *c*_*i*_ the feature *t*_*k*_ contains:

(3) $$\begin{array}{c} \text{MI}\left(t_{k},c_{i}\right)=\log\frac{P\left(t_{k},c_{i}\right)}{P\left(t_{k}\right)P\left(c_{i}\right)}, \end{array}$$

where *P*(*t*_*k*_, *c*_*i*_) is the probability that feature *t*_*k*_ occurs in category *c*_*i*_.

### 2.4. Document Frequency (DF)

Document Frequency calculates the number of documents in which a feature occurs. The basic idea is that the rare terms are not useful for category prediction and maybe degrade the global performance [7]. The larger the number of the documents containing the feature *t*_*k*_ in the category *c*_*i*_ is, the more predictable information for category *c*_*i*_ the feature *t*_*k*_ possesses [1]. The Document Frequency of a term is calculated as follows:

(4) $$\begin{array}{c} \text{DF}\left(t_{k},c_{i}\right)=P\left(t_{k}c_{i}\right). \end{array}$$

### 2.5. Improved Gini Index (GINI)

The Gini index was originally developed for the best split in decision tree induction [15]. In order to utilize it in text categorization with multiclass setting, the original Gini index was improved by Shang et al. [27]. The improved Gini index measures the purity of feature *t*_*k*_ toward a category *c*_*i*_. The bigger the value of purity is, the better the feature is. The formula of the improved Gini index is defined as follows:

(5) $$\begin{array}{c} \text{Gini}\left(t_{k}\right)=\sum_{i}P{\left(t_{k}\;|\;c_{i}\right)}^{2}P{\left(c_{i}\;|\;t_{k}\right)}^{2}, \end{array}$$

where *P*(*t*_*k*_ | *c*_*i*_) is the probability that the feature *t*_*k*_ occurs in category *c*_*i*_ and *P*(*c*_*i*_ | *t*_*k*_) refers to the conditional probability that the feature *t*_*k*_ belongs to the category *c*_*i*_ when the feature *t*_*k*_ occurs.

### 2.6. DIA Association Factor (DIA)

DIA association factor [1, 28] is used to evaluate the conditional probability of a document being assigned to category *c*_*i*_ when it contains the term *t*_*k*_. It determines the significance of the term *t*_*k*_ for the category *c*_*i*_. The bigger the DIA of the term *t*_*k*_ with respect to category *c*_*i*_ is, the more significant for category *c*_*i*_ the term *t*_*k*_ is. The DIA association factor is defined by

(6) $$\begin{array}{c} \text{DIA}\left(t_{k},c_{i}\right)=P\left(c_{i}\;|\;t_{k}\right), \end{array}$$

where *P*(*c*_*i*_ | *t*_*k*_) refers to the conditional probability that feature *t*_*k*_ belongs to category *c*_*i*_ when the feature *t*_*k*_ occurs.

### 2.7. Comprehensive Measurement Feature Selection (CMFS)

CMFS [11] is a new feature-selection algorithm proposed in our previous research work, in which the significance of a term both in intercategory and intracategory is comprehensively measured. The experimental results show that the CMFS can significantly improve the performance of the classifier:

(7) $$\begin{array}{c} \text{CMFS}\left(t_{k},c_{i}\right)=P\left(t_{k}\;|\;c_{i}\right)P\left(c_{i}\;|\;t_{k}\right). \end{array}$$

### 2.8. Orthogonal Centroid Feature Selection (OCFS)

The Orthogonal Centroid Feature Selection selects features optimally according to the objective function implied by the Orthogonal Centroid algorithm [17, 18]. The centroid of each category and entire training set are used to calculate the score of the term. The score of a term *t*_*k*_ is calculated as follows:

(8) $$\begin{array}{c} \text{OCFS}\left(t_{k}\right)=\overset{\left|C\right|}{\underset{i=1}{\sum }}\frac{n_{i}}{n}{\left(m_{i}^{k}-m^{k}\right)}^{2}, \end{array}$$

where *n*_*i*_ is the amount of documents in the category *c*_*i*_, *n* is the amount of documents in the training set, *m*_*i*_^*k*^ is the *k*th element of the centroid vector *m*_*i*_ of class *c*_*i*_, *m* ^*k*^ is the *k*th element of the centroid vector *m* of entire training set, and |*C*| refers to the number of categories in the corpus.

### 2.9. Deviations from Poisson Feature Selection (DFPFS)

The Poisson distribution has been successfully used to select the effective query words in information retrieval. The DFPFS is derivedfrom Poisson distribution and measures the degree at which a feature deviates from the Poisson distribution [15]. The farther a feature departs from Poisson distribution, the more effective it is. Conversely, if a feature can be predicted by Poisson distribution, then it is poor:

(9) DFPFS(ti,Cj)=(aij−a^ij)2a^ij+(bij−b^ij)2b^ij +(cij−c^ij)2c^ij+(dij−d^ij)2d^ij,a^ij=n(Cj){1−exp⁡(−λi)},  b^ij=n(Cj)exp⁡(−λi),c^ij=n(C¯j){1−exp⁡(−λi)},  d^ij=n(C¯j)exp⁡(−λi),λi=FiN,

where *F*_*i*_ is the total frequency of term *t*_*i*_ in all messages and *n*(*C*_*j*_) and $n({\bar{C}}_{j})$ are the numbers of messages which occur in *C*_*j*_ and are absent from *C*_*j*_, respectively.

## 3. Algorithms

### 3.1. Motivation

Prior to feature selection for text categorization, a term-to-category matrix [11], in which rows are the features and columns are category vector, must be generated. In fact, the term-to-category matrix is the foundation of most feature-selection algorithms. All the feature-selection algorithms only consider the term frequency of a feature occurring in a given category and do not take the influence of the imbalance problem into consideration.  Table 1 shows 5 features in term-to-category matrix for top 10 categories of Reuters-21578 corpus. The number in the parentheses indicates the sum of documents in the corresponding category. It can be seen from Table 1 that categories C1 and C4 have significantly more training documents than other categories, and, hence, the term frequency of many features appearing in these two categories is significantly higher than their frequency in other categories; for example, the total term frequency of five features occurring in categories C1 and C4 is 3853 and 5700, respectively. However, we think that the term frequency of a feature occurring in one majority category cannot suggest the essence of the feature in this category; the number of one feature occurring in one minority category cannot reflect the truth of the feature in this category. Based on this observation, a scheme which can eliminate the influence of the imbalance problem for feature-selection algorithms is proposed in this paper.

### 3.2. The Improved Scheme

Feature selection contains three steps. The first step is to calculate the significance of a particular feature *t*_*k*_ over a given category *c*_*i*_(FS(*t*_*k*_, *c*_*i*_)). FS(*t*_*k*_, *c*_*i*_) is the local significance of the feature. The second step is to combine the category-specific scores of each feature into one score (FS(*t*_*k*_)). FS(*t*_*k*_) is the global significance of the feature [7]. The last step is to rank all features in the training set according to the global significance of each feature and then select the top *k* significant features as a new feature subset. To eliminate the negative influence of the imbalance problem, the local significance of feature *t*_*k*_ can be calculated using

(10) $$\begin{array}{c} \text{FS}\left(t_{k},c_{i}\right)=\frac{\text{FS}\left(t_{k},c_{i}\right)}{P\left(c_{i}\right)}, \end{array}$$

where *P*(*c*_*i*_) is the probability of category *c*_*i*_ occurring in the entire training set. Two alternate ways can be used to calculate the value of *P*(*c*_*i*_). One is to use the number of documents to calculate the probability *P*(*c*_*i*_); the other is to use the amount of all features occurring in category *c*_*i*_ to calculate the probability *P*(*c*_*i*_). In this paper, (12) is used:

(11) $$\begin{array}{c} P\left(c_{i}\right)=\frac{n_{i}}{n}, \end{array}$$

(12) $$\begin{array}{c} P\left(c_{i}\right)=\frac{tf_{i}}{\sum_{j=1}^{\left|C\right|}tf_{j}}, \end{array}$$

where *n* is the total number of documents in the entire training set; *n*_*i*_ is the sum of the documents in category *c*_*i*_; *tf*_*i*_ is the amount of features occurring in category *c*_*i*_; |*C*| is the number of the categories.

There are two alternate ways that calculate the global significance of one feature based on the local significance. In one way the average value of one feature over all categories will be taken as the global value. The formula for the average way is shown in (13). In the other way the maximum value of one feature over all categories will be regarded as the global score. The formula for the maximum way is shown in (14). In order to weaken the influence of the imbalance problem, we substitute (15) and (16) for (13) and (14) in this paper:

(13) $$\begin{array}{c} \text{FS}{\left(t_{k}\right)}_{\text{avg}}=\overset{\left|\text{C}\right|}{\underset{i=1}{\sum }}P\left(c_{i}\right)\text{FS}\left(t_{k},c_{i}\right), \end{array}$$

(14) $$\begin{array}{c} \text{FS}{\left(t_{k}\right)}_{\max}=\;\overset{\left|C\right|}{\underset{i=1}{\text{MAX}}}\left\{\text{FS}\left(t_{k},c_{i}\right)\right\}, \end{array}$$

(15) $$\begin{array}{c} \text{FSX}{\left(t_{k}\right)}_{\text{avg}}=\overset{\left|\text{C}\right|}{\underset{i=1}{\sum }}\text{FS}\left(t_{k},c_{i}\right), \end{array}$$

(16) $$\begin{array}{c} \text{FSX}{\left(t_{k}\right)}_{\max}=\;\overset{\left|C\right|}{\underset{i=1}{\text{MAX}}}\left\{\frac{\text{FS}\left(t_{k},c_{i}\right)}{P\left(c_{i}\right)}\right\}. \end{array}$$

Based on the idea proposed in this paper, the feature-selection algorithms listed in Section 2 can be improved.  Table 2 shows the improved formula of nine existing feature-selection algorithms in Section 2. Since the category-specific score of GINI is not provided in the literature about the GINI algorithm, the extension version of local feature selection for GINI is not listed in Table 2. The category-specific score of OCFS is not described in the literature either; however, it can be deduced from the formula of OCFS that (*m*_*i*_^*k*^ − *m* ^*k*^)^2^ is the local significance of the feature *t*_*k*_.

## 4. Experiment Setup

### 4.1. Classifiers

In this paper, both NB and SVM are used to make a comparison before and after nine existing feature-selection methods are improved, respectively.

NB [4] is an excellent algorithm for text categorization. It is based on the assumption that a term occurring in a document is independent of other terms. There are two commonly used models for Bayesian classifier: one is the multivariate Bernoulli model; the other is the multinomial model which is used in this paper.

SVM, which was developed by Drucker et al. [3] for spam categorization and applied to text categorization by Joachims [29], is a higher efficient classifier in text categorization. In our study, LIBSVM toolkit [30] is used and the options for LIBSVM are assigned the default value.

### 4.2. Datasets

Three benchmark datasets (Reuters-21578, WebKB, and 20-Newsgroups) were used to evaluate the performance of the proposed method in our experiments. In the preprocessing step, all words were converted to lower case, punctuation marks were removed, stop lists were used, and no stemming was used. Document Frequency of a term was used in the text representation, and 10-fold validation was adopted in this paper.

The 20-Newsgroups dataset is one of the standard corpora for text categorization. It contains 19997 Newsgroup postings, and all documents were assigned evenly to 20 different UseNet groups.

21578 stories in Reuters-21578 dataset, which are from the Reuters newswire, are nonuniformly divided into 135 categories. In this paper, the top 10 categories are used.

The WebKB, which is a collection of web pages from four different college web sites, contains 8282 web pages. All web pages are nonuniformly assigned to 7 categories. In this paper, four categories (“course,” “faculty,” “project,” and “student”) are used.

### 4.3. Performance Measures

The text categorization effectiveness is usually measured using the F1, accuracy, and AUC [1, 31]. F1 measure is a combined effectiveness measure determined by “precision” and “recall.” Precision is the conditional probability that the decision is correct when a random document is classified under a specific category. Recall is the conditional probability that the decision is taken when a random document ought to be classified under a specific category. The formulas of the precision and recall for the category *c*_*i*_ are defined as

(17) $$\begin{array}{c} P_{i}=\frac{TP_{i}}{TP_{i}+FP_{i}},\;\;R_{i}=\frac{TP_{i}}{TP_{i}+FN_{i}}, \end{array}$$

where *TP*_*i*_ is the amount of the documents that are correctly classified to category *c*_*i*_; *FP*_*i*_ is the amount of the documents that are misclassified to category *c*_*i*_; *FN*_*i*_ is the amount of the documents which belong to category *c*_*i*_ and are misclassified to other categories. For evaluating performance average across categories, the microaveraging was used in our experiments. The microprecision and microrecall may be obtained as

(18) Pmicro=TPTP+FP=∑i=1|C|TPi∑i=1|C|(TPi+FPi),Rmicro=TPTP+FN=∑i=1|C|TPi∑i=1|C|(TPi+FNi),

where |*C*| is the number of the categories. The micro-F1 and accuracy are defined in the following way:

(19) F1micro=2PmicroRmicroPmicro+Rmicro,Accuracy=TP+TNTP+TN+FP+FN.

The receiver operating characteristics (ROC) curve provides a powerful method to visualize performance of the classifier [22]. The area under the ROC curve (AUC) has become a wide measurement of performance of supervised classification rules. However, the simple form of AUC is only applicable to the case of two classes [32]. To calculate the multiclass AUC, the method proposed by Provost and Dominigos [33] is used in our experiments. First, the ROC curve of each class versus all other classes [34] is generated and their respective AUC is measured. Second, the expected AUC is the weighted average of all the AUCs.

## 5. Results

### 5.1. The Experimental Results on 20-Newsgroups

Tables 3 and 4 show the performance comparison of nine improved and existing feature-selection algorithms in terms of micro-F1 and AUC on 20-Newsgroups, respectively. It can be seen from Tables 3 and 4 that the performance of improved version of CHI, DIA, MI, DF, GINI, CMFS, and OCFS is significantly superior to that of the old version. Although the micro-F1 and AUC of NB based on the improved version of IG are inferior to that of existing version of IG, the performance of SVM based on the improved version of IG is superior to that of IG. Moreover, the performance of the improved version of Deviation from Poisson Feature Selection is inferior to that of the old version.

**Figure 1:**
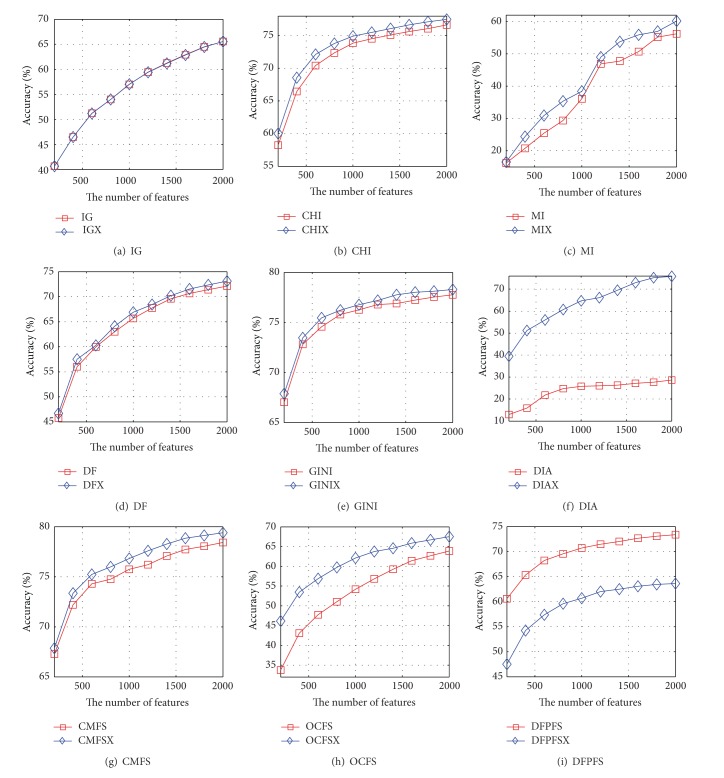
The accuracy curves of NB based on nine pairs of feature-selection methods on 20-Newsgroups, respectively.

**Figure 2:**
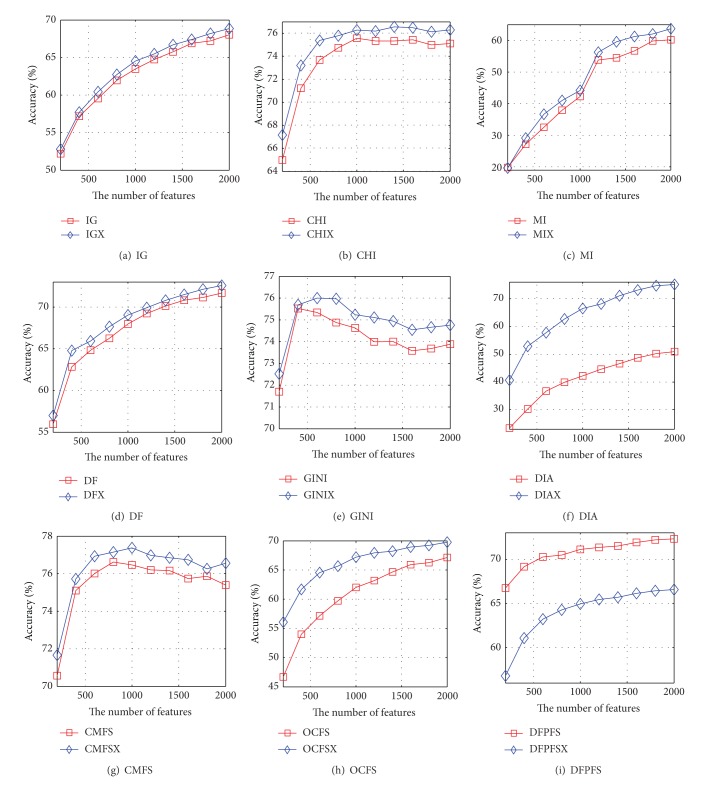
The accuracy curves of SVM based on nine pairs of feature-selection methods on 20-Newsgroups, respectively.

Figures 1 and 2 show the accuracy curves of NB and SVM based on nine pairs of feature-selection methods with 20-Newsgroups, respectively. The value of *x*-axis in Figures 1 and 2 is the number of features selected by different feature-selection algorithms.  Figure 1 indicates that the accuracy curve of NB based on CHIX, MIX, DFX, GINIX, DIAX, CMFSX, and OCFSX is significantly higher than that of CHI, MI, DF, GINI, DIA, CMFS, and OCFS. The extent of the performance growth of DIAX is the highest and the highest growth rate is 165 percent. The accuracy curves of NB based on IGX and IG completely coincide with each other in shape. However, the curve of NB based on DFPFSX is lower than that of DFPFS. It can be seen from Figure 2 that the curve of SVM based on improved version is higher than that of existing version except for DFPFS.

### 5.2. The Experimental Results on Reuters-21578

Table 5 shows the comparison of nine improved and existing feature-selection methods in terms of micro-F1 on Reuters-21578, respectively. It can be seen from Table 5 that the micro-F1 of NB based on CHIX, DFX, DIAX, OCFSX, and DFPFSX is superior to that of CHI, DFX, DIA, OCFS, and DFPFS. The micro-F1 of NB based on IGX is superior to that of IG when the number of selected features is 800 or 1200, respectively. The micro-F1 of NB based on MIX is superior to that of MI when the number of selected features is 400, 1600, or 2000, respectively. The micro-F1 of NB based on GINIX is superior to that of GINI when the number of selected features is 400, 800, 1600, or 2000, respectively. The micro-F1 of NB based on CMFSX is superior to that of CMFS when the number of selected features is 400, 800, 1200, or 1600, respectively. The micro-F1 of SVM based on IGX, CHIX, DFX, GINIX, DIAX, CMFSX, OCFSX, and DFPFSX is significantly superior to that of IG, CHI, DF, GINI, DIA, CMFS, OCFS, and DFPFS. The micro-F1 of SVM based on MIX is superior to that of MI when the number of selected features is 1200, 1600, or 2000, respectively.

Table 6 indicates that the AUCs of SVM based on the improved feature-selection methods on Reuters-21578 are almost superior to that of the nine existing methods. Although some of AUCs of NB on Reuters-21578 based on the improved feature-selection methods are inferior to that of the existing feature-selection algorithms, there is no significant difference between them.

**Figure 3:**
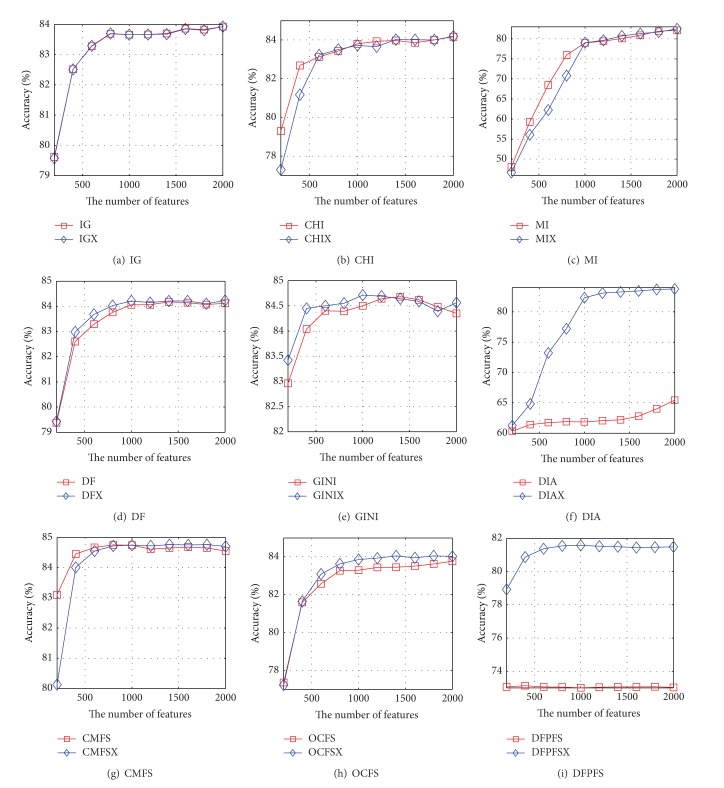
The accuracy curves of NB based on nine pairs of feature-selection methods on Reuters-21578, respectively.

**Figure 4:**
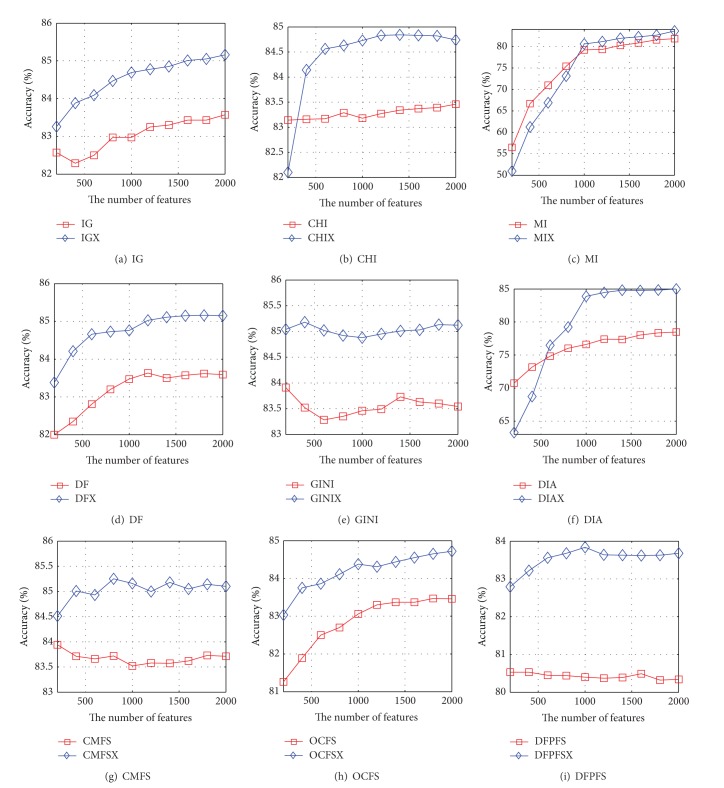
The accuracy curves of SVM based on nine pairs of feature-selection methods on Reuters-21578, respectively.

Based on nine pairs of feature-selection methods and Reuters-21578, the accuracy curves of the NB and SVM are shown in Figures 3 and 4, respectively. It can be seen from Figure 3 that the accuracy curve of NB based on IGX almost coincides with that of IG. The accuracy curve of NB based on CHIX is higher than that of CHI except that the number of features is 200, 400, 1200, or 1400. The accuracy curve of NB based on MIX is higher than that of MI when the number of features is greater than 1000. The accuracy curves of NB based on DFX, DIAX, and DFPFSX are higher than those of DF, DIA, and DFPFS, but the growth rate of performance of DFX is quite small. The accuracy performance of NB based on GINIX is superior to that of GINI except that the number of features is 1400, 1600, or 1800. The accuracy performance of NB based on CMFSX is superior to that of GINI except that the number of features is 200, 400, or 800. The accuracy curve of NB based on OCFSX is higher than that of OCFS when the number of features is greater than 400.  Figure 4 indicates that the accuracy curves of SVM based on IGX, DFX, DIAX, CMFSX, OCFSX, and DFPFSX are significantly higher than those of IG, DF, DIA, CMFS, OCFS, and DFPFS, respectively. When the number of features is greater than 200, the accuracy curve of SVM based on CHIX is higher than that of CHI. The performance of SVM based on MIX is superior to that of MI when the number of features is greater than 1000. The accuracy curve of SVM based on DIAX is higher than that of DIA when the number of selected features is greater than 400.

### 5.3. The Experimental Results on WebKB

Table 7 indicates the comparison of nine improved and existing feature-selection methods with respect to micro-F1 measure on WebKB, respectively. It can be seen from Table 7 that the micro-F1 of NB based on CHIX, DFX, GINIX, DIAX, CMFSX, OCFSX, and DFPFSX is significantly superior to that of CHI, DF, GINI, DIA, CMFS, OCFS, and DFPFS, respectively; the micro-F1 of NB based IGX is superior to that of IG when the number of selected features is 400 or 2000; the micro-F1 of NB based on MIX is superior to that of MI when the number of the selected features is greater than 200. The micro-F1 of SVM based on IGX, CHIX, DFX, GINIX, CMFSX, OCFSX, and DFPFSX is significantly superior to that of IG, CHI, DF, GINI, CMFS, OCFS, and DFPFS.

Table 8 lists the AUCs of NB and SVM on WebKB based on nine improved and existing feature-selection algorithms, respectively. The AUCs of SVM based on the improved feature-selection methods are superior to that of the existing methods except for the DIAX and MIX. The AUCs of NB based on IGX is higher than that of IG when the number of selected features is 400 or 2000. The AUC of NB based on DIAX is superior to that of DIA when the number of features is 1200, 1600, or 2000.

**Figure 5:**
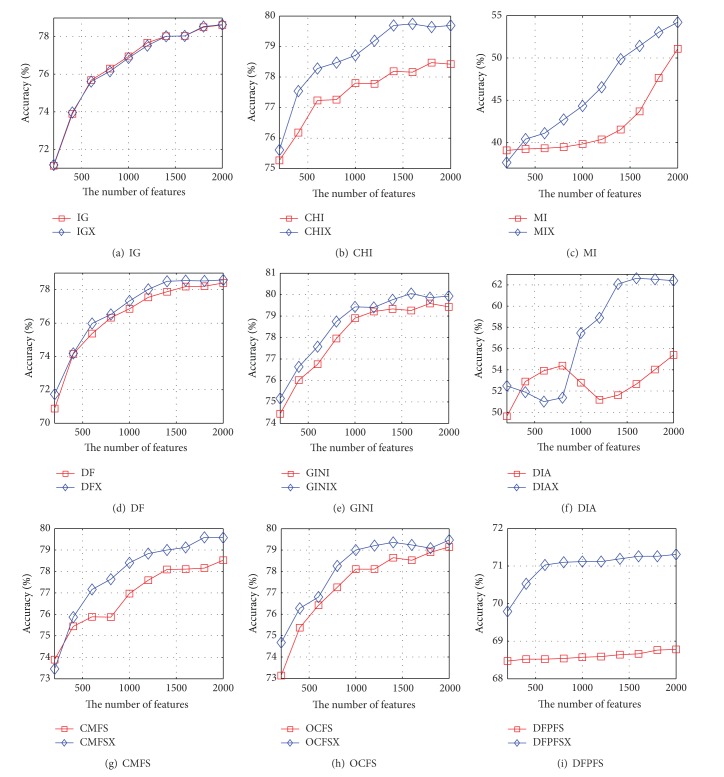
The accuracy curves of NB based on nine pairs of feature-selection methods on WebKB, respectively.

**Figure 6:**
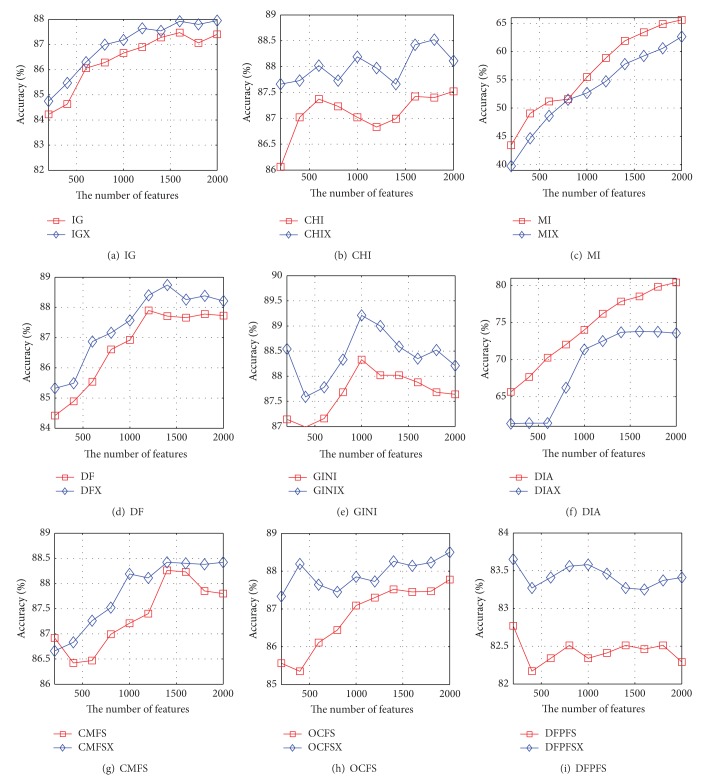
The accuracy curves of SVM based on nine pairs of feature-selection methods on WebKB, respectively.

Figures 5 and 6 show the accuracy curves of NB and SVM based on nine pairs of feature-selection methods on WebKB, respectively. The accuracy curve of NB based on IGX is very close to that of IG. The accuracy curves of NB based on DFX, MIX, DFX, GINIX, CMFSX, OCFSX, and DFPFSX are significantly higher than those of DF, MI, DF, GINI, CMFS, OCFS, and DFPFS. When the number of features is greater than 800, the accuracy of NB based DIAX is superior to that of DIA. The accuracy curves of SVM based on IGX, CHIX, DFX, GINIX, CMFSX, OCFSX, and DFPFSX are significantly higher than those of IG, CHI, DF, GINI, CMFS, OCFS, and DFPFS. However, the accuracy curves of SVM based on MIX and DIAX are lower than those of MI and DIA, respectively.

## 6. Discussion

**Figure 7:**
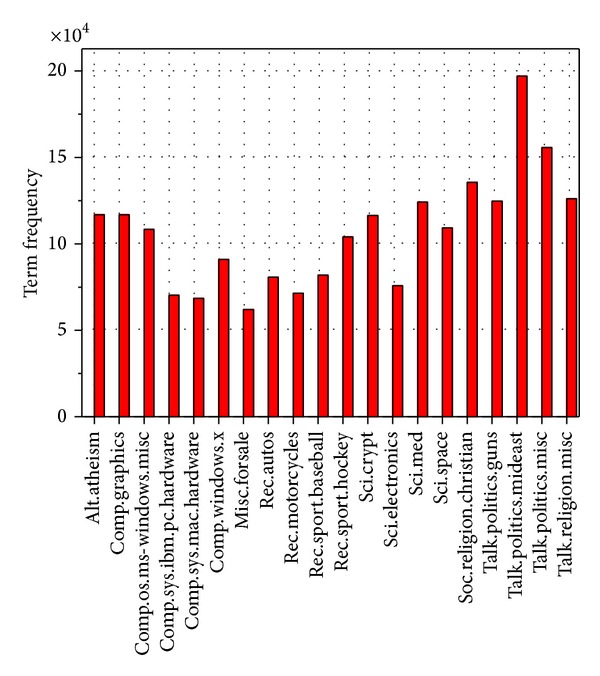
The total number of term frequency of each category in 20-Newsgroups.

Because the amount of documents in every category is equal, 20-Newsgroup is a balance dataset in the view of the number of documents in each category. However, the length of different documents is not identical and the number of terms contained in each document is also different.  Figure 7 shows the total number of term frequency of each category in 20-Newsgroups dataset. It can be seen from Figure 7 that the sum of term frequency of the category “*talk.politics.mideast*” is maximum; the total number of term frequency of the category “*misc.forsale*” is minimum. Hence, it can be seen from Table 2 and Figures 1 and 2 that the performance of the improved feature-selection algorithms, which alleviate the effect of the imbalance factor, is significantly superior to that of existing feature-selection methods.

The expected cross-entropy (ECE) is a feature-selection algorithm used by Zhang and Qiu [35]. The formula of expected cross-entropy is defined by (20). It can be concluded from the experiments that the performance of ECE is superior to that of most of feature-selection algorithms.  Table 9 lists the accuracy comparison of NB between ECE and nine existing feature-selection algorithms on 20-Newsgroups when the number of selected features is 400, 800, 1200, 1600, or 2000. It can be seen from Table 9 that the performance of ECE is superior to that of CHI, DF, IG, MI, OCFS, DIA, and DPFFS and inferior to that of GINI and CMFS. By analyzing of the formula of ECE, we found that the imbalance factor (*P*(*c*_*i*_)) has been considered by ECE; it is the reason why the ECE is more effective than others:

(20) $$\begin{array}{c} \text{ECE}\left(t_{k},c_{i}\right)=P\left(t_{k}\right)\overset{\left|C\right|}{\underset{i=1}{\sum }}P\left(c_{i}\;|\;t_{k}\right)\log\frac{P\left(c_{i}\;|\;t_{k}\right)}{P\left(c_{i}\right)}. \end{array}$$

The time complexity of the improved feature-selection algorithm is higher than that of old version. The reason is that the cost of calculating the prior probability (*P*(*c*_*i*_)) in the improved feature-selection method has been taken into account. There are two ways to calculate the time complexity based on the formula of *P*(*c*_*i*_). We assume that the size of vector space is |*V*| and the number of categories is |*C*|. If the *P*(*c*_*i*_) is evaluated with the amount of documents in every category, the time complexity of *P*(*c*_*i*_) is *O*(|*C*|). If *P*(*c*_*i*_) is evaluated with the sum of term frequency of all features in every category, the cost of *P*(*c*_*i*_) is *O*(|*C*| · |*V*|).

To learn more about our experiments, readers can visit the web site (http://pan.baidu.com/s/1y8z7 K).

## 7. Conclusion 

Feature-selection algorithm is designed to measure the significance of a feature for categorization on the basis of the balance dataset. Though most datasets are balanced in the view of the number of documents in every category, they are imbalanced in the view of the number of features in every category. Thus the traditional feature-selection algorithm does not achieve the best performance due to the adverse effect of the imbalance factor in the corpus. In this paper, we proposed an improved scheme which can weaken the adverse effect caused by the imbalance factor in the corpus. In our experiments, nine well-known feature-selection algorithms are improved using the scheme proposed in this paper. The experimental results indicate that the improved scheme can effectively enhance the performance of text categorization.

## Figures and Tables

**Table 1:** The term-to-category feature appearance matrix.

Features	C1 (2369)	C2 (237)	C3 (578)	C4 (3964)	C5 (582)	C6 (478)	C7 (717)	C8 (286)	C9 (486)	C10 (283)
Billion	345	60	251	1828	110	344	461	26	992	36
Company	2128	6	303	1515	22	6	14	42	24	9
April	622	113	121	1578	304	210	243	121	202	156
Bank	487	5	67	527	24	780	1138	19	141	2
Oil	271	17	2018	252	48	28	36	210	94	32

Total	3853	201	2760	5700	508	1368	1892	418	1453	235

**Table 2:** The improved formula of nine feature-selection algorithms listed in Section 2.

	Local feature selection	Global feature selection
IG	$\text{IGX}\left(t_{k},c_{i}\right)=\frac{1}{p(c_{i})}\left\{\underset{c\in \left\{c_{i},{\overset{-}{c}}_{i}\right\}}{\sum }\underset{t\in \left\{t_{k},{\overset{-}{t}}_{k}\right\}}{\sum }P\left(t,c\right)\log\frac{P(t,c)}{P(t)P(c)}\right\}$	$\text{IGX}\left(t_{k}\right)=\overset{\left|C\right|}{\underset{i=1}{\sum }}\left\{\underset{c\in \left\{c_{i},{\overset{-}{c}}_{i}\right\}}{\sum }\underset{t\in \left\{t_{k},{\overset{-}{t}}_{k}\right\}}{\sum }P(t,c)\log\frac{P(t,c)}{P(t)P(c)}\right\}$

CHI	$\text{CHIX}\left(t_{k},c_{i}\right)=\frac{N{\left(a_{ki}d_{ki}-b_{ki}c_{ki}\right)}^{2}}{\left(a_{ki}+b_{ki})(a_{ki}+c_{ki})(b_{ki}+d_{ki})(c_{ki}+d_{ki})P(c_{i}\right)}$	$\text{CHIX}\left(t_{k}\right)=\overset{\left|C\right|}{\underset{i=1}{\sum }}\frac{N{\left(a_{ki}d_{ki}-b_{ki}c_{ki}\right)}^{2}}{\left(a_{ki}+b_{ki})(a_{ki}+c_{ki})(b_{ki}+d_{ki})(c_{ki}+d_{ki}\right)}$

MI	$\text{MIX}\left(t_{k},c_{i}\right)=\frac{1}{P(c_{i})}\log\frac{P(t_{k},c_{i})}{P(t_{k})P(c_{i})}$	$\text{MIX}\left(t_{k}\right)=\overset{\left|C\right|}{\underset{i=1}{\sum }}\log\frac{P(t_{k},c_{i})}{P(t_{k})P(c_{i})}$

DF	$\text{DFX}\left(t_{k},c_{i}\right)=\frac{P(t_{k}|c_{i})}{P(c_{i})}$	$\text{DFX}\left(t_{k}\right)=\overset{\left|C\right|}{\underset{i=1}{\sum }}P(t_{k}|c_{i})$

GINI	—	$\text{GINIX}\left(t_{k}\right)=\overset{\left|C\right|}{\underset{i=1}{\sum }}\frac{P{\left(t_{k}|c_{i}\right)}^{2}P{\left(c_{i}|t_{k}\right)}^{2}}{P(c_{i})}$

DIA	$\text{DIAX}\left(t_{k},c_{i}\right)=\frac{P(c_{i}|t_{k})}{P(c_{i})}$	$\text{DIAX}\left(t_{k}\right)=\overset{\left|C\right|}{\underset{i=1}{\sum }}P(c_{i}|t_{k})$

CMFS	$\text{CMFSX}\left(t_{k},c_{i}\right)=\frac{P(t_{k}|c_{i})P(c_{i}|t_{k})}{P(c_{i})}$	$\text{CMFSX}\left(t_{k}\right)=\overset{\left|C\right|}{\underset{i=1}{\sum }}P(t_{k}|c_{i})P(c_{i}|t_{k})$

OCFS	$\text{OCFSX}\left(t_{k},c_{i}\right)=\frac{{\left(m_{i}^{k}-m^{k}\right)}^{2}}{P(c_{i})}$	$\text{OCFSX}\left(t_{k}\right)=\overset{\left|C\right|}{\underset{i=1}{\sum }}{\left(m_{i}^{k}-m^{k}\right)}^{2}$

DFPFS	DFPFSX(*t*_*i*_, *C*_*j*_) $=\frac{1}{P(c_{j})}\left\{\frac{{\left(a_{ij}-{\hat{a}}_{ij}\right)}^{2}}{{\hat{a}}_{ij}}+\frac{{\left(b_{ij}-{\hat{b}}_{ij}\right)}^{2}}{{\hat{b}}_{ij}}+\frac{{\left(c_{ij}-{\hat{c}}_{ij}\right)}^{2}}{{\hat{c}}_{ij}}+\frac{{\left(d_{ij}-{\hat{d}}_{ij}\right)}^{2}}{{\hat{d}}_{ij}}\right\}$	DFPFSX(*t*_*i*_) $=\overset{\left|C\right|}{\underset{i=1}{\sum }}\left\{\frac{{\left(a_{ij}-{\hat{a}}_{ij}\right)}^{2}}{{\hat{a}}_{ij}}+\frac{{\left(b_{ij}-{\hat{b}}_{ij}\right)}^{2}}{{\hat{b}}_{ij}}+\frac{{\left(c_{ij}-{\hat{c}}_{ij}\right)}^{2}}{{\hat{c}}_{ij}}+\frac{{\left(d_{ij}-{\hat{d}}_{ij}\right)}^{2}}{{\hat{d}}_{ij}}\right\}$

**Table 3:** The comparison of nine improved and existing feature-selection methods with respect to micro-*F*1 measure on 20-Newsgroups for NB and SVM, respectively. The bold values indicate the best performance of the classifier when various feature-selection methods are used, respectively.

	Naïve Bayes					Support vector machines				
	400	800	1200	1600	2000	400	800	1200	1600	2000
IG	**49.55**	**57.76**	**62.88**	**65.99**	**68.34**	58.15	62.53	65.17	67.28	68.45
IGX	49.54	57.76	62.85	65.92	68.33	**59.01**	**63.51**	**66.06**	**67.91**	**69.32**

CHI	67.29	73.26	75.46	76.58	77.61	73.67	76.07	76.26	76.18	75.60
CHIX	**69.48**	**74.66**	**76.39**	**77.46**	**78.35**	**75.14**	**77.09**	**77.22**	**77.27**	**76.96**

MI	23.13	33.80	51.88	55.47	60.50	27.46	38.24	54.21	57.02	60.62
MIX	**27.39**	**39.06**	**53.78**	**59.97**	**63.87**	**29.17**	**41.18**	**56.71**	**61.61**	**64.19**

DF	59.23	66.37	70.32	72.78	74.07	63.57	66.69	69.57	71.23	72.06
DFX	**60.88**	**67.35**	**70.97**	**73.62**	**74.94**	**65.71**	**68.17**	**70.37**	**71.92**	**72.97**

GINI	73.79	76.68	77.83	78.21	78.74	77.13	75.58	74.50	73.99	74.26
GINIX	**74.45**	**77.17**	**78.22**	**78.94**	**79.18**	77.13	**76.75**	**75.68**	**74.97**	**75.16**

DIA	15.45	22.97	25.70	28.51	30.96	33.53	42.42	46.56	50.22	52.19
DIAX	**47.90**	**59.59**	**66.08**	**73.45**	**76.23**	**59.57**	**67.94**	**72.12**	**76.22**	**77.80**

CMFS	73.05	75.68	77.13	78.57	79.17	76.91	77.50	76.80	76.20	75.75
CMFSX	**74.24**	**76.84**	**78.36**	**79.56**	**80.07**	**77.37**	**77.96**	**77.55**	**77.24**	**77.00**

OCFS	46.50	55.37	60.64	64.72	66.87	54.87	60.30	63.59	66.36	67.61
OCFSX	**55.49**	**62.33**	**66.25**	**68.36**	**69.89**	**63.08**	**66.51**	**68.60**	**69.45**	**70.21**

DFPFS	**67.36**	**71.41**	**73.11**	**74.14**	**74.77**	**69.97**	**71.21**	**72.00**	**72.50**	**72.92**
DFPFSX	56.74	61.90	64.15	64.94	65.40	62.15	65.11	66.22	66.91	67.31

**Table 4:** The comparison of nine improved and existing feature-selection methods with respect to AUC on 20-Newsgroups for NB and SVM, respectively. The bold values indicate the best performance of the classifier when various feature-selection methods are used, respectively.

Feature selection	Naïve Bayes					Support vector machines				
	400	800	1200	1600	2000	400	800	1200	1600	2000
IG	**0.7183**	**0.7579**	**0.7864**	**0.8048**	0.8187	0.7746	0.7998	0.8144	0.8257	0.8318
IGX	0.7182	0.7578	0.7863	0.8045	0.8187	**0.7774**	**0.8038**	**0.8183**	**0.8284**	**0.8361**

CHI	0.8234	0.8545	0.8660	0.8717	0.8771	0.8485	0.8671	0.8702	0.8707	0.8690
CHIX	**0.8346**	**0.8621**	**0.8710**	**0.8770**	**0.8815**	**0.8589**	**0.8726**	**0.8747**	**0.8763**	**0.8753**

MI	0.5830	0.6283	0.7205	0.7405	0.7695	0.6165	0.6733	0.7569	0.7721	0.7905
MIX	**0.6020**	**0.6597**	**0.7315**	**0.7677**	**0.7902**	**0.6269**	**0.6894**	**0.7700**	**0.7961**	**0.8089**

DF	0.7685	0.8054	0.8302	0.8454	0.8531	0.8043	0.8224	0.8380	0.8464	0.8509
DFX	**0.7763**	**0.8111**	**0.8338**	**0.8501**	**0.8585**	**0.8146**	**0.8297**	**0.8416**	**0.8500**	**0.8557**

GINI	0.8569	0.8726	0.8778	0.8801	0.8829	0.8711	0.8678	0.8631	0.8609	0.8625
GINIX	**0.8603**	**0.8749**	**0.8800**	**0.8843**	**0.8858**	**0.8720**	**0.8735**	**0.8690**	**0.8660**	**0.8672**

DIA	0.5571	0.6040	0.6107	0.6165	0.6245	0.6331	0.6838	0.7086	0.7302	0.7419
DIAX	**0.7429**	**0.7933**	**0.8219**	**0.8577**	**0.8728**	**0.7519**	**0.8038**	**0.8324**	**0.8585**	**0.8690**

CMFS	0.8536	0.8671	0.8748	0.8828	0.8865	0.8689	0.8769	0.8747	0.8723	0.8705
CMFSX	**0.8598**	**0.8736**	**0.8821**	**0.8887**	**0.8915**	**0.8722**	**0.8798**	**0.8788**	**0.8776**	**0.8766**

OCFS	0.7083	0.7375	0.7892	0.8017	0.8113	0.7962	0.8164	0.8260	0.8301	0.8351
OCFSX	**0.7550**	**0.7882**	**0.8091**	**0.8205**	**0.8290**	**0.7984**	**0.8193**	**0.8313**	**0.8365**	**0.8408**

DFPFS	**0.8175**	**0.8398**	**0.8499**	**0.8561**	**0.8600**	**0.8377**	**0.8446**	**0.8492**	**0.8521**	**0.8543**
DFPFSX	0.7591	0.7873	0.8000	0.8056	0.8086	0.7952	0.8119	0.8181	0.8218	0.8240

**Table 5:** The comparison of nine improved and existing feature-selection methods with respect to micro-*F*1 measure on Reuters-21578 for NB and SVM, respectively. The bold values indicate the best performance of the classifier when various feature-selection methods are used, respectively.

Feature selection	Naïve Bayes					Support vector machines				
	400	800	1200	1600	2000	400	800	1200	1600	2000
IG	**62.09**	64.60	64.76	**65.11**	65.22	61.31	62.19	62.66	62.59	62.88
IGX	62.07	**64.61**	**64.77**	65.10	65.22	**64.82**	**65.89**	**66.56**	**66.66**	**67.01**

CHI	62.89	64.02	64.96	64.92	65.35	62.85	63.12	62.96	62.93	62.73
CHIX	**64.66**	**65.70**	**66.00**	**66.07**	**65.94**	**67.36**	**67.29**	**67.28**	**67.13**	**66.82**

MI	34.65	**54.87**	**59.71**	61.58	62.77	**43.02**	**51.67**	56.94	58.98	60.11
MIX	**39.55**	51.46	59.64	**61.84**	**63.43**	39.47	51.35	**61.41**	**63.44**	**64.59**

DF	62.41	64.09	64.99	65.36	65.51	61.07	62.75	62.69	62.70	62.82
DFX	**63.99**	**65.38**	**65.65**	**65.92**	**65.94**	**65.90**	**66.65**	**67.05**	**67.01**	**67.58**

GINI	65.13	65.82	**66.64**	66.16	65.78	63.42	63.17	63.07	62.95	62.71
GINIX	**66.43**	**66.45**	66.39	**66.54**	**66.49**	**67.44**	**67.18**	**66.99**	**67.11**	**67.20**

DIA	30.10	30.82	31.49	32.85	37.60	45.06	48.87	51.05	51.85	53.04
DIAX	**48.11**	**57.71**	**63.69**	**64.23**	**64.95**	**49.79**	**59.15**	**65.73**	**66.39**	**66.96**

CMFS	66.03	66.52	66.38	66.66	**66.64**	63.73	63.52	63.14	63.06	62.97
CMFSX	**66.94**	**67.21**	**66.87**	**66.84**	66.59	**67.48**	**67.41**	**67.10**	**67.27**	**67.45**

OCFS	60.90	63.43	64.41	64.39	64.96	60.28	61.49	62.69	62.52	62.69
OCFSX	**63.91**	**65.62**	**65.56**	**65.65**	**65.85**	**65.82**	**66.11**	**66.19**	**66.55**	**66.92**

DFPFS	57.20	57.04	57.03	57.05	57.03	61.56	61.39	61.27	61.44	61.17
DFPFSX	**62.55**	**63.71**	**63.67**	**63.40**	**63.40**	**64.92**	**65.42**	**65.37**	**65.46**	**65.53**

**Table 6:** The comparison of nine improved and existing feature-selection methods with respect to AUC on Reuters-21578 for NB and SVM, respectively. The bold values indicate the best performance of the classifier when various feature-selection methods are used, respectively.

Feature selection	Naïve Bayes					Support vector machines				
	400	800	1200	1600	2000	400	800	1200	1600	2000
IG	**0.8978**	0.9068	0.9073	**0.9088**	0.9093	0.9005	0.9050	0.9065	0.9079	0.9086
IGX	0.8977	0.9068	0.9073	0.9087	0.9093	**0.9083**	**0.9124**	**0.9143**	**0.9159**	**0.9168**

CHI	**0.8988**	0.9055	**0.9093**	0.9091	0.9112	0.9053	0.9071	0.9066	0.9070	0.9079
CHIX	0.8864	**0.9058**	0.9074	**0.9101**	**0.9116**	**0.9090**	**0.9126**	**0.9143**	**0.9144**	**0.9138**

MI	0.6923	**0.8521**	0.8799	0.8902	0.8979	**0.7957**	**0.8541**	0.8810	0.8904	0.8974
MIX	**0.7282**	0.8256	**0.8815**	**0.8928**	**0.9007**	0.7357	0.8341	**0.8897**	**0.8970**	**0.9070**

DF	0.8977	0.9075	0.9098	0.9107	0.9107	0.9012	0.9060	0.9091	0.9086	0.9090
DFX	**0.9008**	**0.9091**	**0.9105**	**0.9111**	**0.9116**	**0.9103**	**0.9138**	**0.9159**	**0.9169**	**0.9167**

GINI	0.9082	0.9119	0.9135	**0.9138**	0.9123	0.9083	0.9068	0.9075	0.9088	0.9083
GINIX	**0.9112**	**0.9129**	**0.9139**	0.9135	**0.9135**	**0.9165**	**0.9147**	**0.9152**	**0.9157**	**0.9164**

DIA	0.7005	0.7146	0.7249	0.7334	0.7565	**0.8501**	0.8673	0.8750	0.8790	0.8811
DIAX	**0.7884**	**0.8654**	**0.9040**	**0.9066**	**0.9088**	0.7954	**0.8778**	**0.9124**	**0.9143**	**0.9159**

CMFS	**0.9109**	**0.9141**	0.9133	0.9139	0.9134	0.9095	0.9094	0.9084	0.9087	0.9094
CMFSX	0.9071	0.9135	**0.9143**	**0.9147**	**0.9144**	**0.9150**	**0.9171**	**0.9153**	**0.9156**	**0.9162**

OCFS	**0.8983**	**0.9074**	0.9088	0.9092	**0.9104**	0.9027	0.9029	0.9046	0.9057	0.9075
OCFSX	0.8914	0.9065	**0.9091**	**0.9095**	0.9102	**0.9068**	**0.9094**	**0.9111**	**0.9127**	**0.9136**

DFPFS	0.8159	0.8157	0.8159	0.8161	0.8162	0.8875	0.8869	0.8865	0.8871	0.8863
DFPFSX	**0.8828**	**0.8882**	**0.8884**	**0.8880**	**0.8883**	**0.9032**	**0.9066**	**0.9064**	**0.9062**	**0.9067**

**Table 7:** The comparison of nine improved and existing feature-selection methods with respect to micro-*F*1 measure on WebKB for NB and SVM, respectively. The bold values indicate the best performance of the classifier when various feature-selection methods are used, respectively.

Feature selection	Naïve Bayes					Support vector machines				
	400	800	1200	1600	2000	400	800	1200	1600	2000
IG	71.36	**74.17**	**75.84**	**76.61**	77.46	83.52	85.66	86.10	86.80	86.77
IGX	**71.64**	73.96	75.64	76.57	**77.48**	**84.10**	**86.29**	**86.76**	**87.27**	**87.33**

CHI	71.92	74.16	74.65	76.19	76.93	85.88	86.52	86.08	86.89	86.89
CHIX	**74.98**	**76.28**	**77.39**	**78.57**	**78.83**	**86.69**	**86.97**	**87.26**	**87.63**	**87.34**

MI	**33.96**	34.63	36.32	40.71	49.58	**44.96**	**48.60**	**57.23**	**61.75**	**64.44**
MIX	33.69	**38.31**	**44.49**	**51.00**	**54.30**	38.37	46.86	50.56	56.63	60.85

DF	71.04	73.74	75.82	76.87	77.23	83.84	85.87	87.27	87.04	87.05
DFX	**71.93**	**74.48**	**76.57**	**77.52**	**77.55**	**84.51**	**86.28**	**87.73**	**87.64**	**87.59**

GINI	72.13	75.58	77.68	77.84	78.42	86.17	86.81	87.09	87.13	86.90
GINIX	**73.75**	**76.96**	**78.06**	**78.80**	**78.97**	**86.61**	**87.52**	**88.20**	**87.56**	**87.44**

DIA	47.75	48.40	46.06	48.77	51.00	**62.66**	**69.13**	**74.84**	**76.95**	**79.03**
DIAX	**50.11**	**53.23**	**60.99**	**63.86**	**63.68**	56.42	63.42	69.97	71.73	71.36

CMFS	72.14	73.22	75.62	76.81	77.33	85.64	86.06	86.72	**87.63**	87.17
CMFSX	**73.37**	**75.89**	**77.63**	**78.06**	**78.70**	**86.04**	**86.66**	**87.29**	87.54	**87.75**

OCFS	73.07	75.23	76.46	77.09	78.02	84.39	85.64	86.49	86.84	87.04
OCFSX	**73.79**	**76.52**	**78.05**	**78.27**	**78.53**	**87.19**	**86.86**	**86.85**	**87.44**	**87.78**

DFPFS	65.17	64.74	64.65	64.66	65.33	80.69	81.46	81.27	81.26	80.28
DFPFSX	**67.96**	**68.81**	**68.37**	**68.58**	**68.83**	**81.83**	**82.08**	**82.11**	**82.00**	**82.01**

**Table 8:** The comparison of nine improved and existing feature-selection methods with respect to AUC on WebKB for NB and SVM, respectively. The bold values indicate the best performance of the classifier when various feature-selection methods are used, respectively.

Feature selection	Naïve Bayes					Support vector machines				
	400	800	1200	1600	2000	400	800	1200	1600	2000
IG	0.8051	**0.8253**	**0.8369**	**0.8399**	0.8442	0.8933	0.9044	0.9093	0.9128	0.9120
IGX	**0.8055**	0.8242	0.8357	0.8398	**0.8444**	**0.8973**	**0.9080**	**0.9134**	**0.9153**	**0.9151**

CHI	0.8214	0.8312	0.8365	0.8402	0.8423	0.9090	0.9114	0.9095	0.9131	0.9133
CHIX	**0.8345**	**0.8416**	**0.8478**	**0.8521**	**0.8520**	**0.9130**	**0.9137**	**0.9158**	**0.9191**	**0.9170**

MI	0.5009	0.5030	0.5113	0.5419	0.6082	**0.6357**	**0.6618**	**0.7137**	**0.7478**	**0.7620**
MIX	**0.5237**	**0.5505**	**0.5925**	**0.6359**	**0.6593**	0.5681	0.6359	0.6653	0.7024	0.7310

DF	0.8041	0.8242	0.8344	0.8404	0.8422	0.8952	0.9078	0.9157	0.9140	0.9143
DFX	**0.8051**	**0.8262**	**0.8382**	**0.8432**	**0.8439**	**0.8981**	**0.9103**	**0.9181**	**0.9174**	**0.9168**

GINI	0.8177	0.8358	0.8463	0.8476	0.8492	0.9105	0.9144	0.9171	0.9155	0.9138
GINIX	**0.8229**	**0.8420**	**0.8480**	**0.8534**	**0.8533**	**0.9123**	**0.9179**	**0.9229**	**0.9184**	**0.9174**

DIA	**0.6252**	**0.6372**	0.6058	0.6192	0.6465	**0.7867**	**0.8151**	**0.8420**	**0.8575**	**0.8696**
DIAX	0.5700	0.5931	**0.6111**	**0.6279**	**0.6511**	0.5791	0.6142	0.6473	0.6842	0.7052

CMFS	0.8132	0.8216	0.8354	0.8397	0.8430	0.9067	0.9098	0.9123	0.9181	0.9152
CMFSX	**0.8183**	**0.8351**	**0.8445**	**0.8475**	**0.8513**	**0.9079**	**0.9123**	**0.9165**	**0.9185**	**0.9190**

OCFS	0.8189	0.8349	0.8436	0.8453	0.8490	0.9106	0.9041	0.9119	0.9160	0.9158
OCFSX	**0.8244**	**0.8401**	**0.8480**	**0.8488**	**0.8508**	**0.9151**	**0.9112**	**0.9141**	**0.9168**	**0.9191**

DFPFS	0.7504	0.7507	0.7511	0.7517	0.7525	0.8735	0.8750	0.8743	0.8741	0.8729
DFPFSX	**0.7723**	**0.7770**	**0.7772**	**0.7782**	**0.7786**	**0.8815**	**0.8824**	**0.8819**	**0.8806**	**0.8809**

**Table 9:** The accuracy comparison of ECE with nine feature-selection algorithms when the NB is used on 20-Newsgroups. The numbers in the parentheses are the difference of accuracy of the corresponding feature-selection algorithm from that of ECE.

Feature selections	400	800	1200	1600	2000
ECE	70.81 (—)	74.54 (—)	75.51 (—)	76.73 (—)	77.16 (—)
CHI	66.44 (−4.37)	72.36 (−2.18)	74.54 (−0.97)	75.62 (−1.11)	76.66 (−0.50)
DF	56.01 (−14.8)	63.02 (−11.52)	67.74 (−7.77)	70.64 (−6.09)	72.10 (−5.06)
GINI	72.81 (+2.00)	75.79 (+1.25)	76.78 (+1.27)	77.22 (+0.49)	77.76 (+0.60)
IG	46.47 (−24.34)	53.99 (−20.55)	59.42 (−16.09)	62.92 (−13.81)	65.55 (−11.61)
MI	20.77 (−50.04)	29.38 (−45.16)	46.90 (−28.61)	50.70 (−26.03)	56.21 (−20.95)
DIA	15.86 (−54.95)	24.76 (−49.78)	26.02 (−49.49)	27.14 (−49.59)	28.64 (−48.52)
CMFS	72.19 (+1.38)	74.76 (+0.22)	76.20 (+0.69)	77.72 (+0.99)	78.43 (+1.27)
OCFS	43.10 (−27.71)	51.05 (−23.49)	56.82 (−18.69)	61.41 (−15.32)	63.87 (−13.29)
DFPFS	65.33 (−5.48)	69.56 (−4.98)	71.48 (−4.03)	72.66 (−4.07)	73.39 (−3.77)
